# Revitalising Aging Oocytes: Echinacoside Restores Mitochondrial Function and Cellular Homeostasis Through Targeting GJA1/SIRT1 Pathway

**DOI:** 10.1111/cpr.70044

**Published:** 2025-04-18

**Authors:** Liuqing Yang, Xinle Lai, Fangxuan Lin, Nan Shi, Xinya Xu, Heng Wang, Xiaotian Li, Dan Shen, Haimo Qian, Xin Jin, Jiayi Chen, Zhongwei Huang, Xing Duan, Qin Zhang

**Affiliations:** ^1^ Department of TCM Gynecology Hangzhou TCM Hospital Affiliated to Zhejiang Chinese Medical University Hangzhou China; ^2^ Research Institute of Women's Reproductive Health Zhejiang Chinese Medical University Hangzhou China; ^3^ Zhejiang Key Laboratory of Precise Protection and Promotion of Fertility Hangzhou China; ^4^ NUS Bia‐Echo Asia Centre of Reproductive Longevity and Equality, Yong Loo Lin School of Medicine, National University of Singapore Singapore Singapore; ^5^ Zhejiang Chinese Medical University Hangzhou China; ^6^ Institute of Molecular and Cell Biology, Agency of Science Research and Technology Singapore Singapore; ^7^ Department of Obstetrics and Gynaecology National University Health Systems Singapore Singapore; ^8^ Key Laboratory of Environmental Medicine Engineering, Ministry of Education School of Public Health, Southeast University Nanjing China

**Keywords:** cellular homeostasis, echinacoside, GJA1/SIRT1 pathway, mitochondrial function, oocyte aging, oxidative stress

## Abstract

As maternal age increases, the decline in oocyte quality emerges as a critical factor contributing to reduced reproductive capacity, highlighting the urgent need for effective strategies to combat oocyte aging. This study investigated the protective effects and underlying mechanisms of Echinacoside (ECH) on aging oocytes. ECH significantly improved cytoskeletal stability and chromosomal integrity, as demonstrated by restored spindle morphology and reinforced F‐actin structures, essential for meiotic progression. It also preserved mitochondrial function by restoring membrane potential and dynamics, reducing ROS levels, and downregulating the DNA damage marker γ‐H2AX, thereby alleviating oxidative stress and enhancing genomic stability. Furthermore, ECH promoted cellular homeostasis through modulation of lipid metabolism, autophagy and lysosomal function. Transcriptomic analyses identified GJA1 as a pivotal mediator of ECH's effects, validated through molecular docking and bio‐layer interferometry. Functional studies showed that inhibiting GJA1 significantly reduced ECH's ability to enhance first polar body extrusion rates, mitochondrial function and antioxidant capacity, validating the critical role of the GJA1/SIRT1 pathway in combating oocyte aging. This study provides novel insights into the mechanisms of oocyte rejuvenation and highlights ECH as a promising therapeutic candidate for addressing age‐related reproductive challenges.

## Graphical abstract

ECH protects senescent oocytes by stabilising the cytoskeleton, restoring mitochondrial function, reducing oxidative stress and maintaining cellular homeostasis, primarily through the GJA1/SIRT1 signalling pathway.

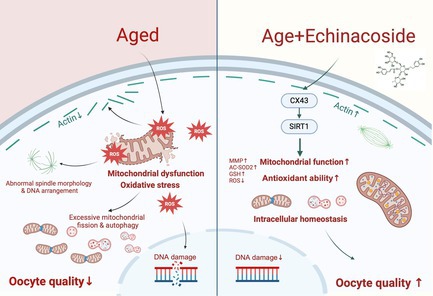

**Abbreviations**
AMA: Advanced maternal age
Anti‐SOD2: Anti‐superoxide dismutase 2
BLI: Bio‐Layer Interferometry
BSA: Bovine Serum Albumin
COCs: Cumulus‐oocyte complexes
DCFH‐DA: 2′,7’‐Dichlorodihydrofluorescein diacetate
DEGs: Differentially Expressed Genes
D‐Gal: D‐Galactose
DPBS: Dulbecco's Phosphate‐Buffered Saline
Drp1: Dynamin‐related protein 1
ECH: Echinacoside
F‐Actin: Filamentous actin
FF: Follicular fluid
Fis1: Fission protein 1
FITC: Fluorescein isothiocyanate
GO: Gene ontology
GSH: Glutathione
H3K9me3: Histone 3 lysine 9 trimethylation
IGF1: Insulin‐like growth factor 1
KEGG: Kyoto encyclopedia of genes and genomes
LC3β: Microtubule‐associated protein 1 light chain 3 beta
LDs: lipid droplets
MD: Molecular dynamics
MFN1: Mitofusin 1
MMP: Mitochondrial membrane potential
MnSOD: Manganese superoxide dismutase
OS: Oxidative stress
PARKIN: Parkin RBR E3 ubiquitin protein ligase
PINK1: PTEN induced putative kinase 1
PPAR: Peroxisome proliferator‐activated receptors
PPI: Protein–Protein Interaction
qPCR: Quantitative polymerase chain reaction
RMSD: Root mean square deviation
ROS: Reactive oxygen species
scRNA‐seq: Single‐cell RNA sequencing
SIRT1: NAD‐dependent protein deacetylase sirtuin‐1
SOD2: Superoxide dismutase 2
SOX9: Transcription factor SOX‐9
TAGLN: Transgelin
TRP: Transient receptor potential
VIM: Vimentin
γH2A.X: γH2A histone family member X

## 1 Introduction

In recent decades, global fertility patterns have significantly transformed due to demographic shifts, socioeconomic factors, and increased educational attainment among women [1, 2]. These changes have contributed to a decline in fertility rates and a trend toward delayed childbearing [3, 4]. Advanced maternal age (AMA), defined as pregnancy at 35 or older, is associated with increased reproductive risks, such as infertility, miscarriage, chromosomal abnormalities, and pregnancy complications [5]. Notably, the proportion of women having their first child after age 35 has risen from 1% to 2%, particularly among those undergoing fertility treatment, where the average age now exceeds 35 years [6, 7]. The miscarriage rate among AMA women is about 25%, rising to 90% for those over 45, with chromosomal abnormalities occurring 2.4 times more frequently than in younger women [8]. Furthermore, the incidence of aneuploid oocytes in women aged 40 is reported to be 20 times higher than in younger women [9]. Chromosomal aneuploidy is considered a central factor driving the age‐related decline in oocyte quality, primarily due to impaired meiotic division and increased spindle abnormalities in aging oocytes. This increased risk directly contributes to higher rates of miscarriage and an elevated likelihood of chromosomal abnormalities during pregnancy [10]. Therefore, the age‐related decline in oocyte quality is a central factor contributing to these reproductive challenges [11]. Despite these challenges, effective strategies to mitigate the age‐related decline in oocyte quality remain limited, making it crucial to develop interventions that improve reproductive outcomes.

Aging leads to a decline in both oocyte quantity and quality, which are primary contributors to age‐related fertility issues [11]. Oocyte maturation requires synchronised nuclear and cytoplasmic maturation, and mitochondrial function plays a crucial role in this process [12]. Mitochondria, as the primary energy producers of the cell, are essential for meiotic progression in oocytes [13, 14]. Dysfunctional mitochondria produce excessive reactive oxygen species (ROS), leading to oxidative stress (OS), mitochondrial DNA damage, and meiotic errors, which further degrade oocyte quality [15]. Addressing mitochondrial dysfunction and OS is therefore critical for anti‐oocyte aging and improving reproductive success.

Echinacoside (ECH), a natural bioactive component derived from various plants such as *Rehmannia glutinosa*, *Cistanche deserticola* , and *Jasminum sambac* , exhibits various notable antioxidant, anti‐inflammatory, and antitumor properties. ECH has attracted research interest as a potential health supplement for anti‐aging due to its significant effects on neurodegenerative diseases, skin aging, and tumours [16, 17, 18, 19]. In terms of reproductive health, ECH has been shown to prevent sperm damage in rats [20], suggesting that it may also hold promise for mitigating age‐related oocyte decline. Interestingly, ECH improves heart failure by attenuating OS and protecting mitochondrial function [21]. Despite these findings, its potential role in countering oocyte aging remains unknown.

This study is the first to explore the potential protective effects of ECH on aging oocytes. Using a galactose (D‐Gal)‐induced aging oocyte model and transcriptomics technology, we deeply investigated the molecular mechanisms by which ECH preserves oocyte quality. The D‐Gal induced aging model is widely used to simulate the physiological aging process, which is closely related to the catalytic action of aldose reductase and the formation of ROS species [22]. Given the physiological similarities between porcine and human oocytes, porcine oocytes were used in this study. Our findings demonstrate that ECH treatment effectively combats oocyte aging, as evidenced by enhanced first polar body extrusion rate, improved spindle morphology, restored mitochondrial function, and maintained cellular homeostasis in aging oocytes. Further single‐cell transcriptome, molecular docking and bio‐layer interferometry assay revealed that ECH exerts its anti‐aging effects primarily through the GJA1/SIRT1 signalling pathway, providing new insights for the protection and intervention of women's reproductive health.

## 2 Materials and Methods

### 2.1 Antibodies and Chemicals

Primary antibodies served in this study were as detailed below: mouse monoclonal anti‐α‐tubulin‐FITC (Sigma, F2168, USA); Rhodamine Phalloidin (Life technologies, R415, USA); Anti‐SOD2/MnSOD (Manganese superoxide dismutase) (acetyl K68) antibody (Abcam, ab137037, UK); PINK1 Polyclonal antibody (Proteintech, 23274‐1‐AP, China); Parkin (PRK8) (proteintch, 66674‐1‐lg); mouse monoclonal LC3β antibody (Santa Cruz, H1821, USA); MFN1 Monoclonal antibody (Proteintech, 66776‐1‐Ig, China); Drp1 Rabbit mAb (Cell Signalling Technology, 8570S, USA); Fis1(Proteintech, 66635‐1, China); and γH2A.X (Cell Signalling Technology, 9718T, USA).

The following secondary antibodies were employed: Coralite 594‐conjugated Goat‐Anti‐Mouse IgG (H + L) (Proteintech, SA00013‐3, China); FITC Goat Anti‐Rabbit IgG (H + L) Antibody (Biodragon, BF05002, China); and Anti‐rabbit IgG Fab2 Alexa Fluor 555 (Cell Signalling, 4413 S, USA). Antibodies used in IF were listed in Table 1.

**TABLE 1:** Antibodies utilised in this study.

Antibody	Source	Catalogue number
Mouse monoclonal anti‐α‐tubulin‐FITC	Sigma	F2168
Rhodamine Phalloidin	Life technologies	R415
Anti‐SOD2/MnSOD (Manganese superoxide dismutase) (acetyl K68) antibody	Abcam	ab137037
PINK1 Polyclonal antibody	Proteintech	23274‐1‐AP
Parkin (PRK8)	Proteintech	66674‐1‐lg
Mouse monoclonal LC3β antibody	Santa Cruz	H1821
MFN1 Monoclonal antibody	Proteintech	66776–1‐Ig
Drp1 Rabbit mAb	Cell Signalling Technology	8570S
Fis1	Proteintech	66635‐1
γH2A.X	Cell Signalling Technology	9718T
Anti‐Histone H3 (trimethyl K9) antibody	ABCAM	ab176916
Coralite 594‐conjugated Goat‐Anti‐Mouse IgG (H + L)	Proteintech	SA00013–3
Anti‐rabbit IgG Fab2 Alexa Fluor 555	Cell Signalling	4413S

### 2.2 Oocyte Culture and Parthenogenetic Activation

Porcine ovaries were sourced from a local abattoir and immediately submerged in sterile saline solution supplemented with 100 IU/mL penicillin and streptomycin for transport to the laboratory. Follicular fluid (FF) was aspirated from follicles measuring 2–8 mm in diameter using a 10 mL syringe with an 18‐gauge needle. The collected FF was centrifuged, and the pellet was rinsed with Dulbecco's Phosphate‐Buffered Saline (DPBS), followed by supernatant removal. Cumulus‐oocyte complexes (COCs) with at least four dense layers of cumulus cells were selected and cultured in TCM‐199 medium (Sigma, M4530, USA).

To induce oocyte aging, D‐Gal (Sigma, G5388, USA) was dissolved in TCM‐199 medium to obtain working concentrations of 25 and 50 mM, following the manufacturer's protocol. Based on preliminary experiments assessing the impact of D‐Gal on polar body extrusion and cumulus expansion, 50 mM was chosen for further studies. ECH (MedChemExpress, HY‐N0020) was also dissolved in TCM‐199 medium containing 50 mM D‐Gal, and a range of concentrations was tested. Ultimately, 100 μM was selected based on its effects on cumulus expansion and polar body extrusion.

Oocytes were divided into three experimental groups: the Control group, cultured in TCM‐199 medium alone; the D‐Gal group, cultured in TCM‐199 medium supplemented with 50 mM D‐Gal; and the D‐Gal + ECH group, cultured in TCM‐199 medium containing 50 mM D‐Gal and 100 μM ECH.

After 42–44 h of maturation, cumulus cells were removed using 1 mg/mL hyaluronidase. Mature oocytes were then positioned between electrodes immersed in fusion solution and activated by two direct current pulses (120 V, 40 μs) using an Eppendorf 22331 system (Eppendorf, Germany). The fusion solution consisted of 3% mannitol containing 0.02499 g/mL CaCl_2_ 2H_2_O and 0.00996 g/mL MgCl_2_·6H_2_O. Activated oocytes were then transferred to PZM‐3 medium supplemented with 5 μg/mL Cytochalasin B for 4 h and subsequently cultured in PZM‐3 at 38.5°C with 5% CO_2_. Cleavage rates were assessed after 24 and 48 h of in vitro culture.

### 2.3 Immunofluorescence Staining and Confocal Microscopy

After 24–28 h of culture, COCs were treated with 0.1% hyaluronidase in TCM‐199 medium, then rinsed. Oocytes were fixed in 4% paraformaldehyde for 30 min, permeabilised with 1% Triton X‐100 for 8 h, and blocked with 1% BSA for 1 h. Primary antibodies (α‐tubulin‐FITC 1:750, Rhodamine Phalloidin 1:750, ac‐SOD2 1:300, PINK1 1:300, Parkin 1:300, LC3β 1:300, MFN1 1:300, Drp1 1:300, P‐DRP1 1:300, Fis1 1:300, γH2A.X 1:400) were applied overnight at 4°C. Secondary antibodies (Alexa Fluor 555, FITC Goat Anti‐Rabbit, Coralite 594, FITC Goat Anti‐Mouse, all at 1:500) were incubated for 1 h. Nuclei were stained with Hoechst 33342 (1:750) for 10 min. Confocal microscopy (FV3000, Olympus) was used for imaging, and ImageJ (NIH) for data analysis.

### 2.4 Detection of Mitochondrial Level and Lysosomes Level

Mitochondrial levels were measured using Mito‐Tracker Red CMXRos (1:400, Beyotime, C1035, China), incubated for 30 min at 38.5°C in a 5% CO_2_ incubator. Lysosomal levels were detected using Lyso‐Tracker Red (75 nM, Beyotime, C1046, China) under the same conditions. Confocal microscopy was used to visualise both.

### 2.5 Detection of ROS and Lipid Droplets Level

ROS levels were detected by incubating oocytes with DCFH‐DA (10 μM, Beyotime, S0033M, China) for 30 min at 38.5°C in a 5% CO_2_ incubator, followed by three washes. Lipid droplets were stained using BODIPY 493/503 (2 μM, GLPBIO, GC42959, USA) under the same conditions. Confocal microscopy was used to visualise ROS and lipid droplets.

### 2.6 Detection of Mitochondrial Membrane Potential

Mitochondrial membrane potential was measured using JC‐1 (Beyotime, C2005, China) after 30 min of incubation at 38.5°C in a 5% CO_2_ incubator. Confocal microscopy was employed for imaging.

### 2.7 Single Cell Transcriptome Sequencing Analysis

The transcriptome analysis of oocytes was conducted using single‐cell RNA sequencing (scRNA‐seq). For each group, three biological replicates (three oocytes per replicate) were collected and lysed. Sequencing was performed by the Beijing Genomics Institute. First‐strand cDNA was synthesised using the Smart‐Seq2 method, followed by PCR amplification to establish the sequencing library. The cDNA was fragmented, end‐repaired, and subjected to poly‐A tailing and adaptor ligation. After library quality control, sequencing was carried out on the Illumina platform, generating paired end reads with a length of 100 bp. Post‐sequencing, low‐quality reads and adapter sequences were removed, and clean reads were used for downstream analysis of Differentially Expressed Genes (DEGs), Gene Ontology (GO) enrichment, and Kyoto Encyclopedia of Genes and Genomes (KEGG) pathway analysis. DEGs between groups were identified using the criteria of |log2FC| > 1 and *p* value ≤ 0.05, followed by GO and KEGG analysis.

### 2.8 Real‐Time Quantitative PCR

Gene expression was analysed by real‐time quantitative PCR (qPCR) using the 2^−ΔΔCT^ method. RNA was extracted from 50 oocytes with the Dynabeads mRNA DIRECT Purification Kit (Invitrogen). cDNA was synthesised using the HiScript II 1st Strand cDNA Synthesis Kit (Vazyme), and qPCR was performed with Hieff qPCR SYBR Green Master Mix (Yeasen). Primer sequences are shown in Table 2.

**TABLE 2:** Sequences of qPCR primers.

Gene	Primers	Primers sequences (5′–3′)
SOX9	Forward	GGCAAACTCTGGAGACTGCTG
	Reverse	GATGGCGTTGGGAGAGATGT
GJA1	Forward	GTTTCCTCTCTCGTCCCACG
	Reverse	ATTCGATTCTGCTCGGCACT
IGF1	Forward	GCCCAAGGCTCAGAAGG
	Reverse	TTTAACAGGTAACTCGTGC
VIM	Forward	GCCGTGGAAGCTGCTAACTA
	Reverse	CCATCTCTGGTCTCAACCGTC
TAGLN	Forward	GGCAGCCCTTTAAACCCCTC
	Reverse	CCATTCTTCAGCCAGACCTGGAA
GAPDH	Forward	GGAAGCTGTGGCGTGATGGC
	Reverse	TTCTCCAGGCGGCAGGTCAG

### 2.9 Protein–Protein Interaction (PPI) Network Construction

The DEGs between groups were input into the STRING database (https://cn.string‐db.org/) for PPI network construction, focusing on *Homo sapiens* proteins with an interaction score > 0.400. The network was visualised using Cytoscape (v3.8.2).

### 2.10 Molecular Dynamic Simulation

Molecular dynamics (MD) simulations were performed on key proteins identified from the PPI network using GROMACS 2020.6. The CHARMM36 force field was applied to parameterise the protein, and ligand topology was generated via the Acpype server (https://www.bio2byte.be/Acpype/). The protein‐ligand complex was modelled in a dodecahedron box and solvated using a three‐point water model. Sodium or chloride ions were added to neutralise the system. Energy minimisation was performed for 50,000 steps at 300 K, followed by 100 ps of NVT and NPT equilibration. A 30 ns production run was executed with a 2.0 fs timestep, and trajectory files were saved every 10 ps. The root mean square deviation (RMSD) of the trajectory was analysed to assess the stability of the complex using GROMACS tools.

### 2.11 Molecular Docking

The 3D structure of the target protein, identified from the PPI network, was retrieved from the RCSB Protein Data Bank (https://www.rcsb.org/). PyMol software (v2.4.0) was used to remove heteroatoms and water molecules from the protein structure. The 2D structure of ECH, the ligand, was downloaded from PubChem (https://pubchem.ncbi.nlm.nih.gov/). Both structures were converted into pdbqt format using AutoDockTools (v1.5.7). Molecular docking was conducted using AutoDock Vina (v1.1.2), and the resulting protein‐ligand complexes were visualised and analysed with PyMol.

### 2.12 Bio‐Layer Interferometry Assay

Bio‐Layer Interferometry (BLI) is ideally suited for the characterisation of protein–protein and protein‐small molecule binding kinetics and binding affinity. *GJA1* was biotinylated and immobilised on an SSA sensor (Cat# 18‐5057). Buffer was added to buffer wells, and ECH (50, 100, 200 and 400 μM) was added to sample wells. The assay was run at 30°C with a shaking speed of 1000 rpm. Baseline, association and dissociation phases were recorded, and the data were analysed using ForteBio Data Analysis software (v12.0) to determine binding kinetics and affinity.

### 2.13 Statistical Analysis

All statistical analyses were performed using GraphPad Prism software (v9.3.1, La Jolla, USA). Data were compared using one‐way analysis of variance followed by Tukey's multiple comparison test. A *p* value of ≤ 0.05 was considered statistically significant.

## 3 Results

### 3.1 Establishment of Aging Oocyte Model and Determination of the Optimal ECH Concentration

**FIGURE 1:**
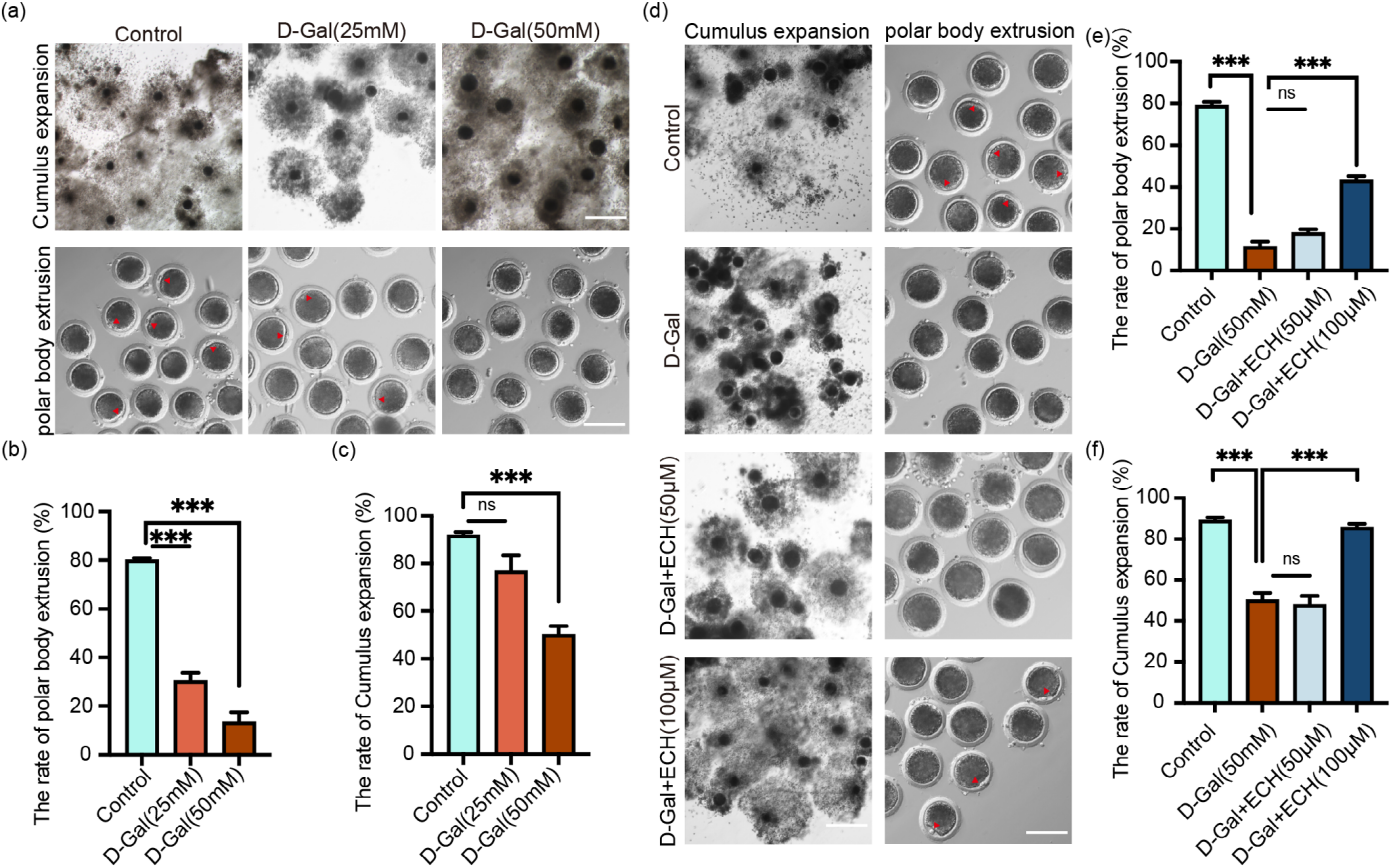
Establishment of aging oocyte model and determination of the optimal ECH concentration. (a) Representative images of COCs expansion and the first polar body extrusion in the Control, D‐Gal (25 mM) and D‐Gal (50 mM) groups, with the red arrow indicating the first polar body. Scale bar, 200 μm. (b) Quantification of first polar body extrusion percentages across the three groups; ***p < 0.001. (c) Quantification of COCs expansion percentages across the three groups; ***p < 0.001. (d) Representative images of cumulus cell expansion and first polar body extrusion in Control, D‐Gal, D‐Gal+ECH (50 μM), and D‐Gal+ECH (100 μM) groups, with the red arrow indicating the first polar body. Scale bar, 200 μm. (e) Quantification of first polar body extrusion percentages across the four groups; ***p < 0.001. (f) Quantification of COCs expansion percentages across the four groups; ***p < 0.001. All data are expressed as the mean ± standard deviation and verified in at least three independent experiments. ECH treatment improved early embryo development in aging oocytes. (a) The representative images of cleavage on the 24 and 48 h of parthenogenetically activated porcine embryos in control, D‐Gal and D‐Gal+ECH groups. Scale Bar = 100 μm. (b) The proportions of embryos at various stages were calculated on the 24 and 48 h in control, D‐Gal and D‐Gal+ECH groups. *p < 0.05; **p < 0.01; ***p < 0.001.

COCs were cultured in vitro with 25 and 50 mM concentrations of D‐Gal to establish an aging oocyte model. As shown in Figure 1a–c, D‐Gal treatment significantly reduced the percentage of expanded COCs in a dose‐dependent manner compared to the control group. The extrusion rate of the first polar body also decreased with increasing D‐Gal concentrations. Based on these results, 50 mM D‐Gal was selected as the optimal concentration for inducing oocyte aging in subsequent experiments.

Subsequently, the effect of ECH on aging oocytes was evaluated. ECH was dissolved in TCM‐199 medium containing 50 mM D‐Gal and administered at concentrations of 50 and 100 μM. Treatment with ECH promoted COCs expansion and significantly increased the first polar body extrusion rate (Figure 1d–f). Notably, the 100 μM ECH group exhibited outcomes closely aligned with the control group, demonstrating its efficacy in reversing D‐Gal‐induced senescence. Consequently, 100 μM ECH was selected for all further studies.

To evaluate the effect of ECH on early embryonic development, porcine parthenogenetic embryos were cultured in PZM‐3 medium. The developmental progression was quantified at 24 and 48 h post‐activation. Statistically significant differences were observed between groups at both the 2‐cell and 4‐cell stages (Figure S1). Remarkably, the 2‐cell development rate in the ECH‐treated group was twice that of the D‐Gal group. This difference became even more pronounced at the 4‐cell stage, underscoring the beneficial effect of ECH on early embryonic development.

### 3.2 ECH Enhances Cytoskeletal and Chromosomal Integrity in Aging Oocytes

**FIGURE 2:**
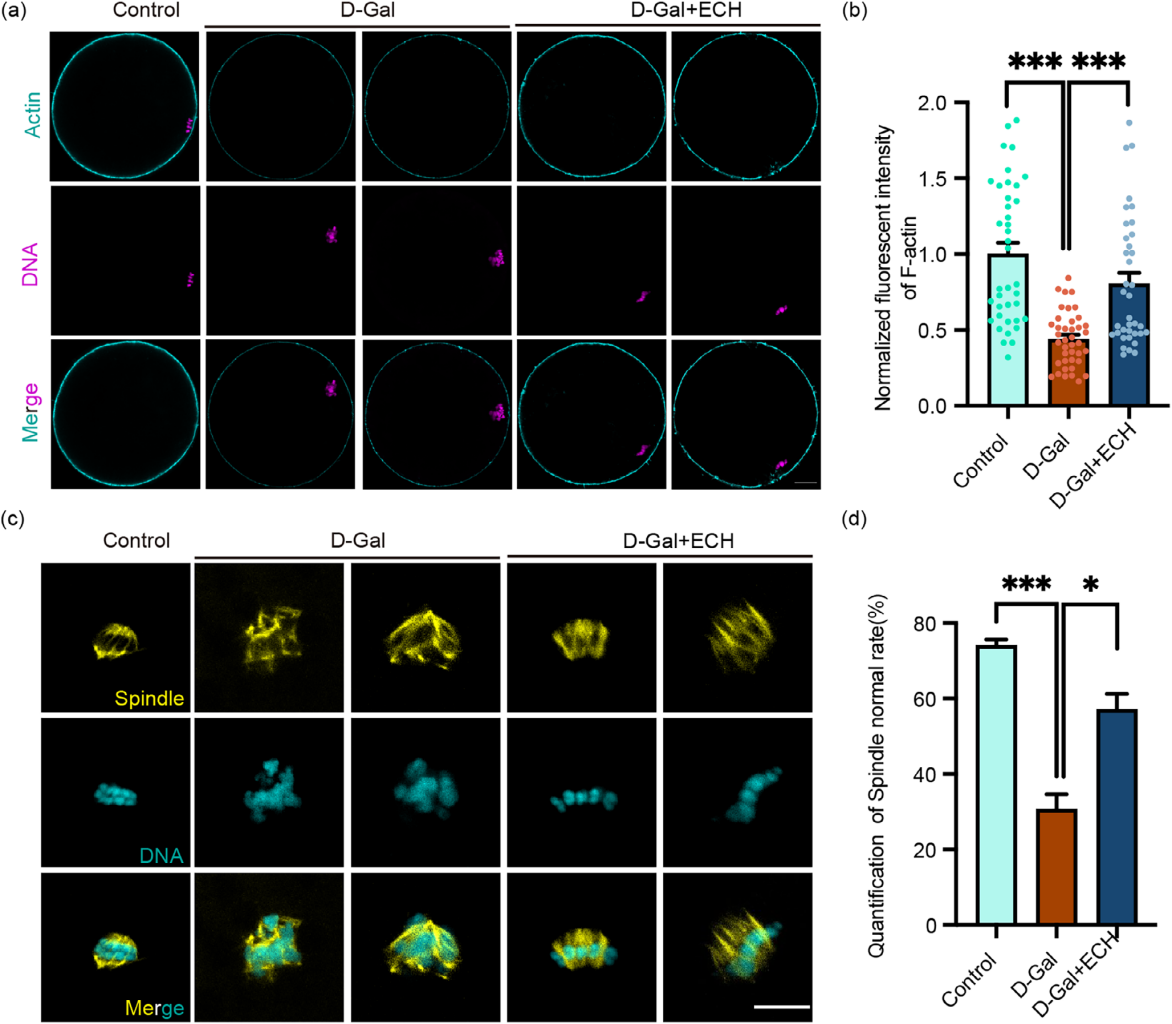
ECH enhances cytoskeletal and chromosomal integrity in aging oocytes. (a) Representative images of rhodamine‐phalloidin staining of F‐actin in the Control, D‐Gal, and D‐Gal+ECH groups. Scale bar, 20 μm. (b) Normalised fluorescence intensity of F‐actin in the Control, D‐Gal and D‐Gal+ECH groups; ***p < 0.001. (c) Representative images of spindle morphology in the Control, D‐Gal, and D‐Gal+ECH groups. Scale bar, 5 μm. (d) Quantification of normal spindle morphology in the Control, D‐Gal, and D‐Gal+ECH groups. ***p < 0.001; *p < 0.05.

To elucidate the mechanisms by which ECH enhances meiotic outcomes in aging oocytes, we analysed F‐actin dynamics and spindle morphology during oocyte maturation. Immunofluorescence staining with rhodamine‐phalloidin revealed that ECH treatment significantly increased F‐actin fluorescence intensity in porcine senescent oocytes compared to the D‐Gal group (Figure 2a,b), indicating that ECH enhanced cytoskeletal integrity essential for maintaining cellular structure and facilitating proper meiotic progression.

Further examination through tubulin immunostaining revealed that oocytes in the D‐Gal group exhibited abnormal spindle structures, characterised by multipolar or nonpolar spindles and misaligned chromosomes—signs of defective microtubule assembly and nucleation (Figure 2c,d). In contrast, ECH treatment largely restored spindle polarity, resulting in a statistically significant increase in the percentage of oocytes with normal spindle morphology compared to the D‐Gal group. This restoration suggests that ECH plays a crucial role in maintaining spindle integrity, thereby ensuring accurate chromosome segregation during meiosis.

### 3.3 ECH Improves Mitochondrial Function and Regulates Mitochondrial Dynamics in Aging Oocytes

**FIGURE 3:**
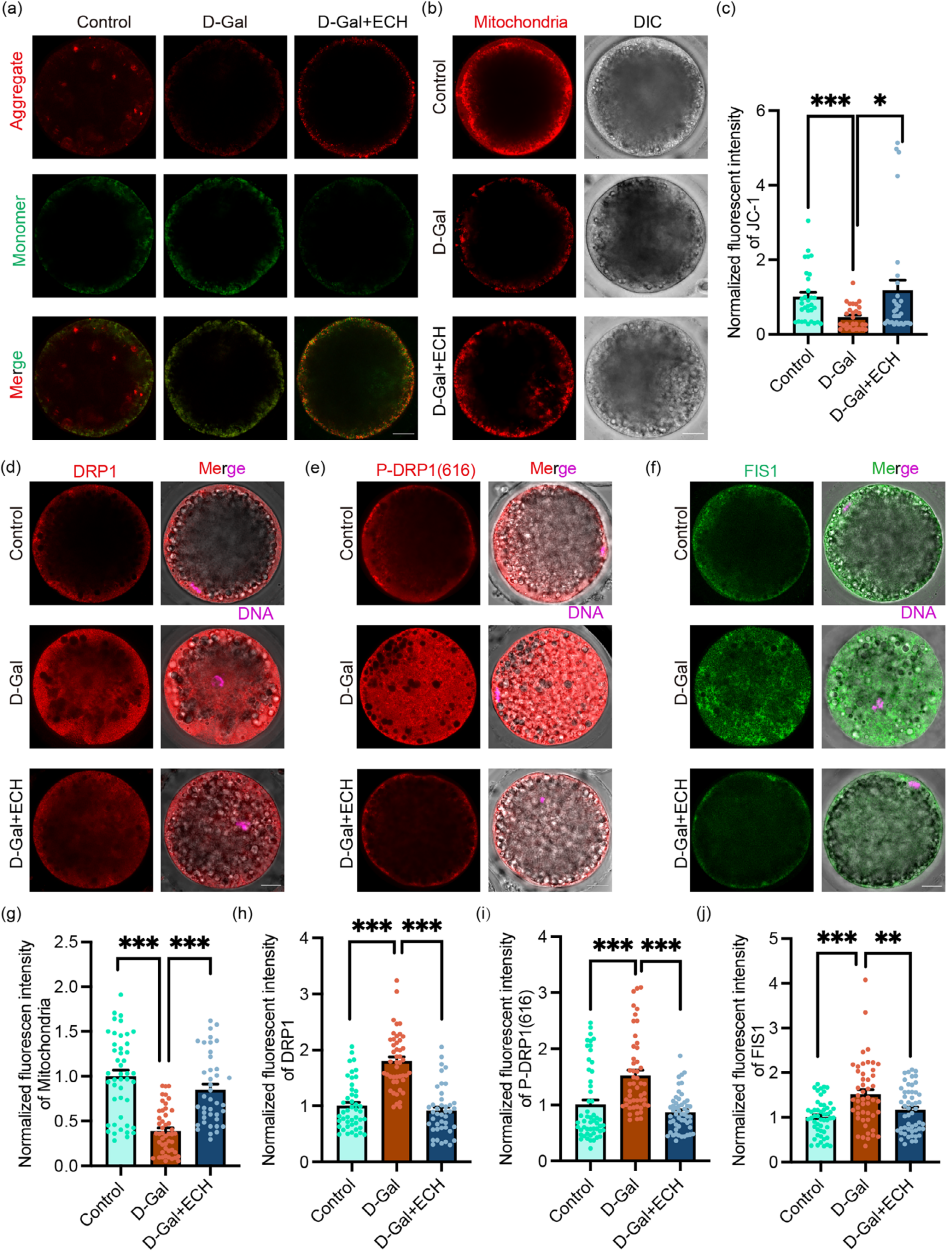
ECH improves mitochondrial function and regulates mitochondrial dynamic in aging oocytes. (a) The representative images of MMP determined by probe JC‐1 in control, D‐Gal and D‐Gal+ECH groups. Scale bar, 20 μm. (b) The representative images of mitochondria level in control, D‐Gal and D‐Gal+ECH groups. Scale bar, 20 μm. (c) The normalised fluorescent intensity of JC‐1 in the three groups; *p < 0.05. (d) Representative images of DRP1 staining in control, D‐Gal and D‐Gal+ECH groups. Scale bar, 20 μm. (e) Representative images of DRP1 (616) staining in control, D‐Gal and D‐Gal+ECH groups. Scale bar, 20 μm. (f) Representative images of FIS1 staining in control, D‐Gal and D‐Gal+ECH groups. Scale bar, 20 μm. (g) The normalised fluorescent intensity of mitochondrial in the three groups; ***p < 0.001. (h) Normalised fluorescent intensity of DRP1 level in control, D‐Gal and D‐Gal+ECH groups; ***p < 0.001. (i) Normalised fluorescent intensity of DRP1 (616) level in control, D‐Gal and D‐Gal+ECH groups; ***p < 0.001. (j) Normalised fluorescent intensity of FIS1 level in control, D‐Gal and D‐Gal+ECH groups; **p < 0.01; ***p < 0.001. All data are expressed as the mean ± standard deviation and verified in at least three independent experiments.

Mitochondrial dysfunction, a critical factor in oocyte aging, is characterised by compromised mitochondrial membrane potential (MMP) and distribution [23]. We utilised JC‐1 staining to assess MMP, revealing a significant reduction in the D‐Gal treated group, which suggests mitochondrial depolarisation. In contrast, ECH treatment markedly restored MMP levels to those comparable with the control group, as evidenced in Figure 3a,c. Furthermore, MitoTracker staining highlighted that D‐Gal disrupted mitochondrial distribution, causing heterogeneous clustering. Conversely, ECH treatment enhanced mitochondrial distribution across the cytoplasm, mirroring the uniform distribution observed in control oocytes (Figure 3b,g).

Additionally, we explored the impact of ECH on mitochondrial dynamics, particularly focusing on proteins governing mitochondrial fission. Immunofluorescence analysis indicated that ECH significantly downregulated the expression of mitochondrial fission proteins DRP1 and its phosphorylated form P‐DRP1(Ser616), as well as FIS1, demonstrating ECH's role in moderating the activities that compromise mitochondrial integrity (Figure 3d–f,h–j).

These findings underscore ECH's efficacy in not only restoring MMP but also in promoting balanced mitochondrial distribution and dynamics, which are essential for maintaining mitochondrial integrity in senescent oocytes. This comprehensive approach highlights the potential of ECH to mitigate the detrimental effects of mitochondrial dysfunction, which is pivotal for improving oocyte health and function.

### 3.4 ECH Reduces and Promotes Cellular Homeostasis in Senescent Oocytes

**FIGURE 4:**
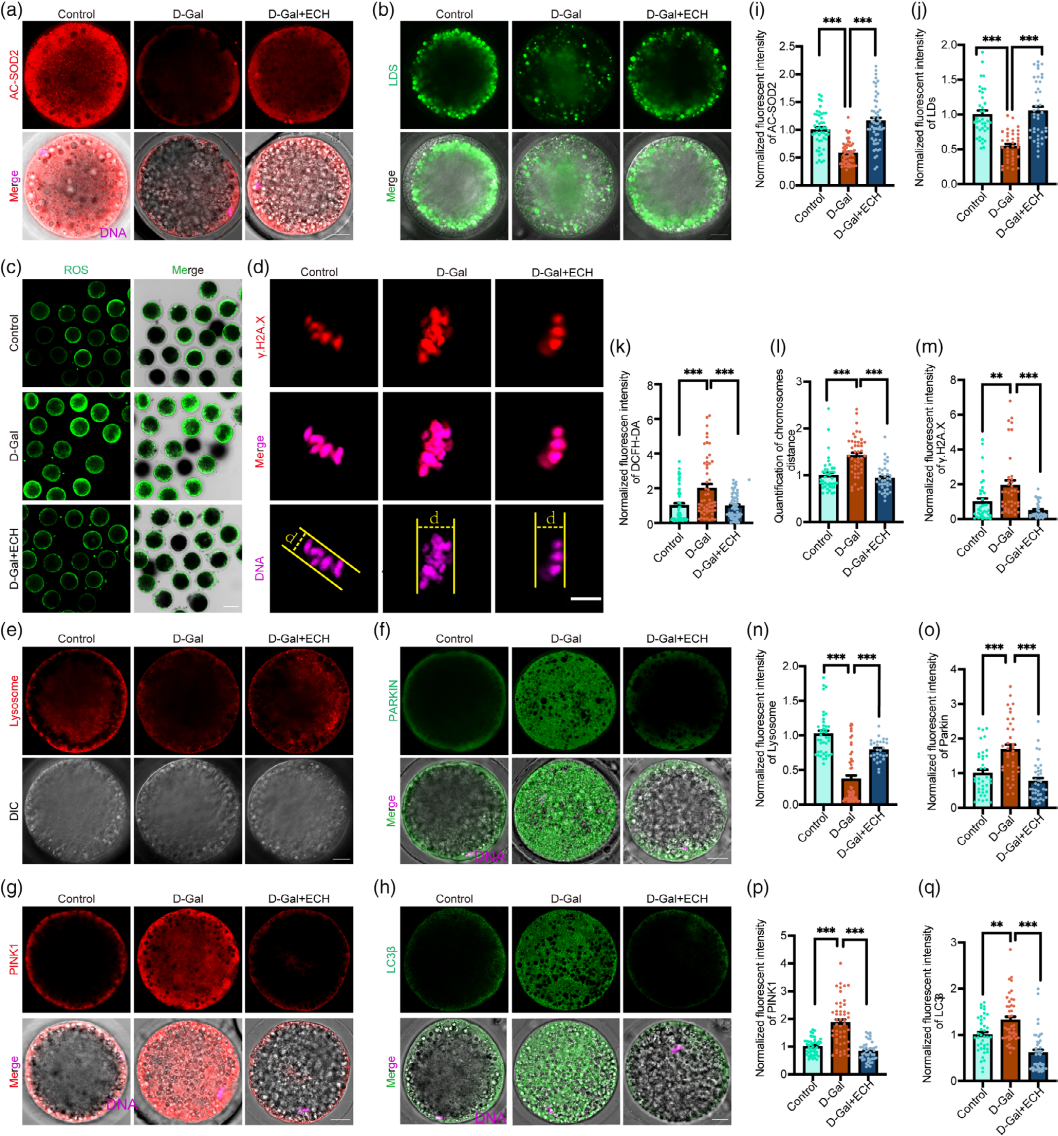
ECH ameliorates oxidative stress in senescent cells. (a) Representative images of AC‐SOD2 distribution in control, D‐Gal and D‐Gal+ECH groups. Scale bar, 20 μm. (b) Representative images of Lipid droplets distribution in control, D‐Gal and D‐Gal+ECH groups. Scale bar, 20 μm. (c) Representative images of DCFH‐DA staining in control, D‐Gal and D‐Gal+ECH groups. Scale bar, 100 μm. (d) The representative images of γ‐H2AX staining and chromosomes distance in the control, D‐Gal, and D‐Gal+ECH groups. Scale bar, 5 μm. (e) Representative images of Lysosomes level in control, D‐Gal and D‐Gal+ECH groups. Scale bar, 20 μm. (f) Representative images of Parkin staining in control, D‐Gal and D‐Gal+ECH groups. Scale bar, 20 μm. (g) Representative images of PINK1 staining in control, D‐Gal and D‐Gal+ECH groups. Scale bar, 20 μm. (h) Representative images of LC3β level in control, D‐Gal and D‐Gal+ECH groups. Scale bar, 20 μm. (i) Normalised fluorescent intensity of AC‐SOD2 in control, D‐Gal and D‐Gal+ECH groups; ***p < 0.001. (j) Normalised fluorescent intensity of LDs in control, D‐Gal and D‐Gal+ECH groups; ***p < 0.001. (k) Normalised fluorescent intensity of DCFH‐DA in control, D‐Gal and D‐Gal+ECH groups; ***p < 0.001. All data are expressed as the mean ± standard deviation and verified in at least three independent experiments. (l) The Quantification of chromosomes distance across the three groups; ***p < 0.001. All data are expressed as mean ± SD and were verified through at least three independent experiments. (m) Normalised γ‐H2AX fluorescent intensity across the three groups. **p < 0.01; *p < 0.05. (n) Normalised fluorescent intensity of Lysosomes level in control, D‐Gal and D‐Gal+ECH groups; ***p < 0.001. (o) Normalised fluorescent intensity of Parkin in control, D‐Gal and D‐Gal+ECH groups; ***p < 0.001. (p) Normalised fluorescent intensity of PINK1 in control, D‐Gal and D‐Gal+ECH groups; ***p < 0.001. (q) Normalised fluorescent intensity of LC3β in control, D‐Gal and D‐Gal+ECH groups. **p < 0.01; ***p < 0.001. All data are expressed as the mean ± standard deviation and verified in at least three independent experiments.

Mitochondrial dysfunction often precipitates an excessive accumulation of ROS, which subsequently triggers OS. We quantified ROS levels using DCFH‐DA staining. Notably, ROS levels were significantly elevated in the D‐Gal treated group compared to controls, while ECH treatment effectively reduced these levels in aging oocytes (Figure 4c). In parallel, we observed a significant rise in acetylation of mitochondrial SOD2, a critical antioxidant enzyme, following ECH treatment (Figure 4a,i). We additionally assessed cellular lipid homeostasis by quantifying lipid droplets (LDs) with Bodipy staining. The fluorescence intensity of LDs was notably reduced in the D‐Gal group, suggesting impaired lipid storage capacity. In contrast, ECH treatment markedly enhanced LD levels, indicative of ameliorated lipid metabolism and diminished ROS‐induced lipid peroxidation (Figure 4b,j).

OS is recognised for causing DNA damage, including single and double‐strand breaks, as well as mutational alterations at specific DNA sites [24]. The extent of DNA damage within senescent oocytes was quantitatively measured using γ‐H2AX staining, a specific marker for detecting double‐strand DNA breaks. Oocytes from the D‐Gal group showed a marked increase in γ‐H2AX fluorescence intensity, indicating heightened DNA damage, as illustrated in Figure 4d,m. In contrast, ECH treatment effectively reduced DNA damage when compared to the D‐Gal condition, underscoring ECH's role in safeguarding against genomic instability.

Lysosomes are crucial organelles responsible for degrading damaged organelles and proteins. Improved lysosomal function facilitates the efficient removal of cellular waste and maintains intracellular homeostasis [25]. Our results showed that aging oocytes treated with ECH exhibited higher lysosomal aggregation and increased LysoTracker fluorescence intensity compared to the D‐Gal group (Figure 4e,n). Furthermore, an abnormal increase in autophagic activity is associated with the cellular stress response [26]. This study showed that autophagy levels were elevated in the D‐Gal group, and ECH treatment significantly reduced LC3β fluorescence intensity (Figure 4h,q), indicating that ECH normalised the autophagic process. Additionally, the PINK1/Parkin signalling pathway, a key regulator of mitochondrial autophagy, showed reduced expression levels of PINK1 and Parkin in the ECH‐treated group (Figure 4f,o,g,p). This reduction suggests that ECH not only regulates general autophagy but also specifically modulates mitophagy, thereby maintaining mitochondrial integrity and further decreasing ROS production.

Together, these findings suggest that ECH reduces OS and promotes cellular homeostasis in aging oocytes by orchestrating the interplay between ROS reduction, lipid metabolism, lysosomal function and autophagic regulation.

### 3.5 Transcriptomic Analysis, Key Gene Validation and Target Identification in ECH‐Rescued Aging Oocytes

**FIGURE 5:**
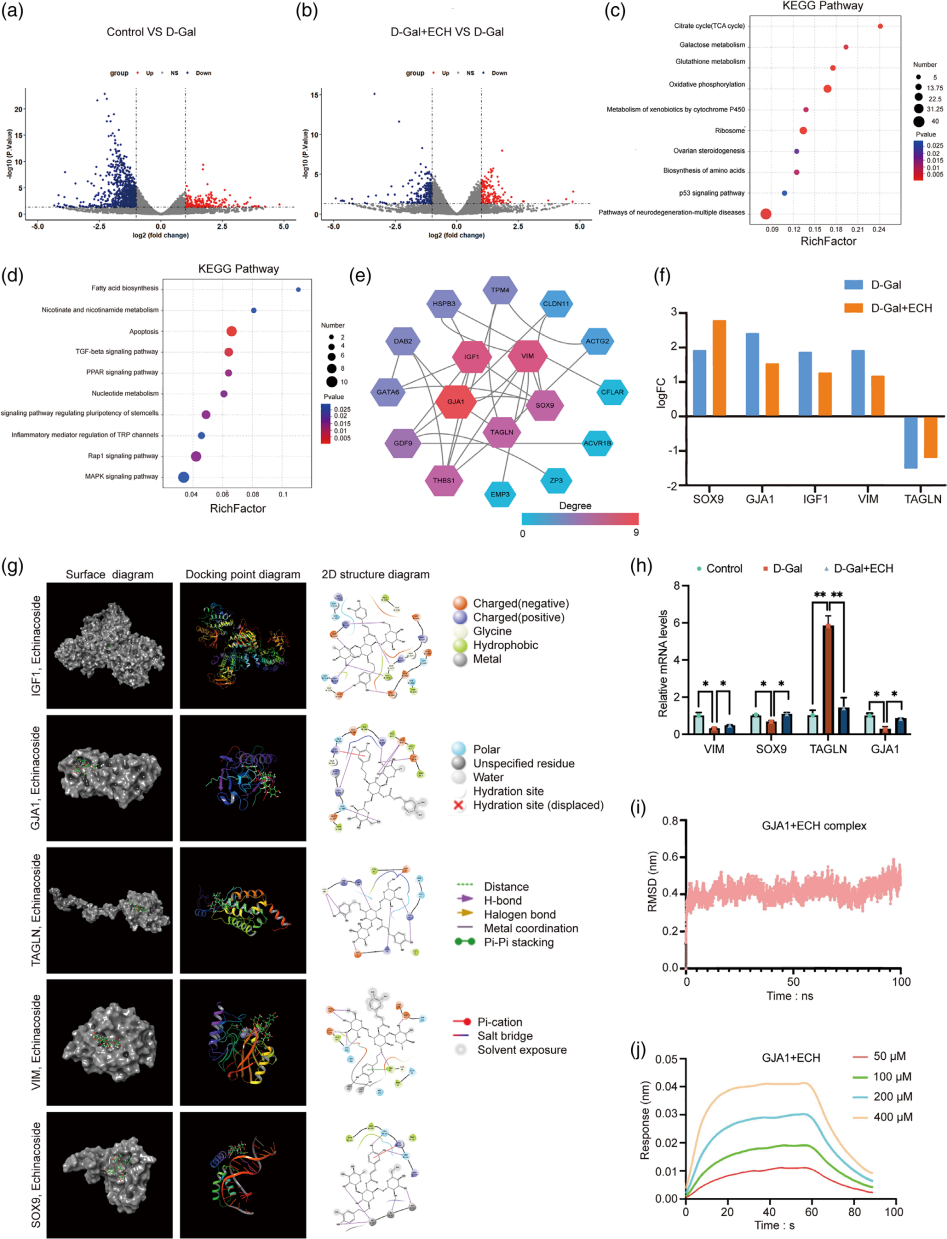
Transcriptome analysis identified potential mechanism by which ECH rescued D‐Gal induced oocyte aging. (a) volcano plot of DEGs of Control versus D‐Gal. (b) volcano plot of DEGs of D‐Gal+ECH versus D‐Gal. (c) KEGG enrichment analysis of DEGs by Control versus D‐Gal. (d) KEGG enrichment analysis of DEGs by D‐Gal+ECH versus D‐Gal. (e) PPI network. (f) logFC of keygene by Control versus D‐Gal and D‐Gal+ECH versus D‐Gal. (g) Molecular docking of 5 hub targets with ECH. (h) The mRNA expression levels of VIM, SOX9, TAGLN and GJA1 were examined by qPCR. *p < 0.05; **p < 0.01. All data are expressed as the mean ± standard deviation and verified in at least three independent experiments. (i) RMSD of GJA1+ECH complex. (g) Binding affinity of GJA1 for ECH determined by bio‐layer interferometry using an Octet RED96e system. Four different drug concentrations including 50, 100, 200 and 400 μM were set. Fitted data are shown as solid lines.

To investigate the molecular mechanisms underlying the protective effects of ECH on D‐Gal‐induced oocyte senescence, we conducted single‐cell transcriptomic analysis. The results identified 1151 DEGs between the control and D‐Gal groups, with 230 genes upregulated and 921 downregulated (Figure 5a). KEGG analysis of these DEGs indicated dysregulation in pathways related to energy metabolism, carbohydrate metabolism, and amino acid metabolism in the senescent oocytes (Figure 5c). In D‐Gal‐induced senescent oocytes treated with ECH, 394 DEGs were identified, including 229 upregulated genes and 165 downregulated genes (Figure 5b). KEGG analysis of these DEGs revealed the involvement of pathways such as inflammatory mediator regulation, TRP channels, apoptosis, and the PPAR signalling pathway in the ECH‐rescued group (Figure 5d).

To determine the key genes of ECH's effect on oocyte aging, we obtained the intersection genes of three groups of DGEs and constructed a PPI network visualised by Cytoscape (Figure 5e). In this PPI network, the degree of each node was used to rank potential key proteins, with higher degrees indicating more significant roles [27]. GJA1, IGF1, VIM, SOX9 and TAGLN were identified as key targets. As shown in Figure 5f, RNA‐seq analysis revealed that GJA1, IGF1, VIM and SOX9 were upregulated in both the control and ECH‐treated groups compared to the D‐Gal‐induced senescence group. Conversely, TAGLN was downregulated in both the control and ECH‐treated groups, indicating that ECH supplementation normalised the transcript levels of these DEGs in D‐Gal‐induced senescent oocytes.

Molecular docking analysis predicted ECH's binding affinities with these key targets by calculating binding energies. Lower binding energy typically indicates a more stable interaction. All calculated binding affinities between ECH and the five targets were below −5 kcal/mol, suggesting strong binding stability (Figure 5g). Notably, ECH formed hydrogen bonds with GJA1 at residues ASP‐110, PRO‐105, and LYS‐96, with IGF1 through bonds at GLU‐33, GLN‐15, GLY‐22, ILE‐43 and VAL‐44, and similarly with SOX9, TAGLN, and VIM at their respective binding residues. To confirm the RNA‐seq findings, qPCR was used to validate the expression patterns of GJA1, VIM, SOX9 and TAGLN. The qPCR results corroborated the transcriptome data, verifying these gene expressions at the mRNA level (Figure 5h).

GJA1, with the highest node degree in the PPI network, suggests it may play a central role in ECH's protective effects against oocyte aging. Given its role in intercellular communication and potential impact on mitochondrial function and cellular homeostasis [28, 29, 30], GJA1 was selected for subsequent experimental validation to investigate its relevance in ECH‐mediated rescue of senescent oocytes. To further assess the stability of the ECH‐GJA1 interaction, MD simulations were performed. The RMSD of the backbone was measured, with fluctuations within 0.2 nm, indicating that the system gradually reached equilibrium (Figure 5i). We also used BLI to probe direct interactions between GJA1 and ECH. Using biotinylated GJA1 immobilised on SSA sensors, four concentrations of ECH (50, 100, 200 and 400 μM) were tested. Sensorgrams showed rapid, dynamic binding of ECH to GJA1, allowing for calculation of the equilibrium binding constant (K_D) as 2.358E‐04, indicating high binding affinity (Figure 5j).

### 3.6 ECH Enhances Senescent Oocyte Function via the GJA1/SIRT1 Pathway

**FIGURE 6:**
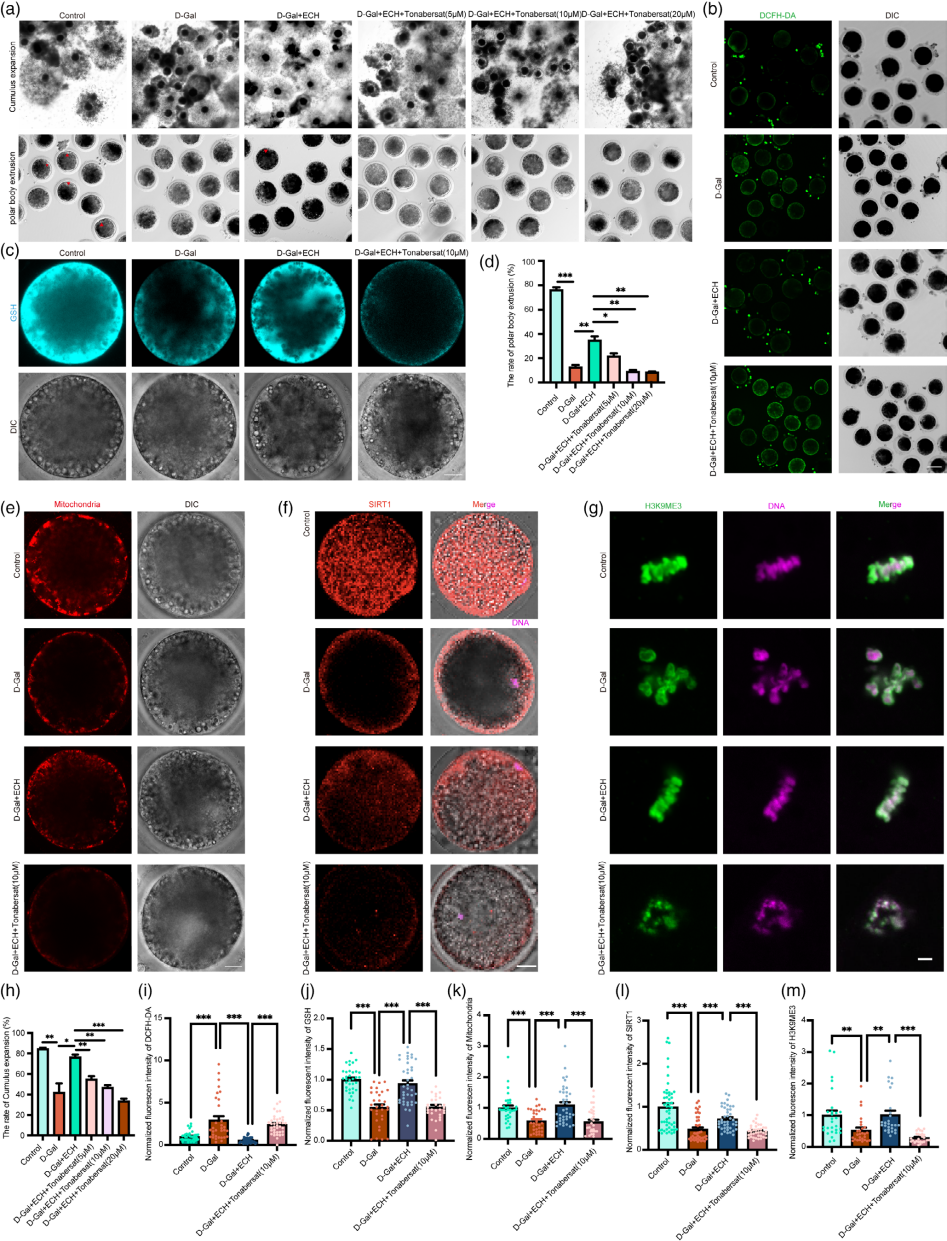
ECH enhances senescent oocyte function via the GJA1/SIRT1 pathway. (a) Representative images of COCs and the first polar body extrusion in the Control, D‐Gal, D‐Gal+ECH, D‐GAl+ECH+Tonabersat (5 μM), D‐GAl+ECH+Tonabersat (10 μM), and D‐GAl+ECH+Tonabersat (20 μM), the red arrow indicates the first polar body. Scale bar, 200 μm. (b) Representative images of DCFH‐DA staining in control, D‐Gal, D‐Gal+ECH and D‐Gal+ECH+Tonabersat (10 μM) groups. Scale bar, 100 μm. (c) Representative images of GSH in control, D‐Gal, D‐Gal+ECH and D‐Gal+ECH+Tonabersat (10 μM) groups. Scale bar, 20 μm. (d) The first polar body extrusion percentage was quantified in six groups. *p < 0.05; **p < 0.01; ***p < 0.001. (e) The representative images of mitochondria distribution in control, D‐Gal, D‐Gal+ECH and D‐Gal+ECH+Tonabersat (10 μM) groups. Scale bar, 20 μm. (f) Representative images of SIRT1 staining in control, D‐Gal, D‐Gal+ECH and D‐Gal+ECH+Tonabersat (10 μM) groups. Scale bar, 20 μm. (g) Representative images of H3K9me3 signals in control, D‐Gal, D‐Gal+ECH, and D‐Gal+ECH+Tonabersat (10 μM) groups. Scale bar, 5 μm. (h) Cumulus cell expansion percentage was quantified in six groups. *p < 0.05; **p < 0.01; ***p < 0.001. All data are expressed as the mean ± standard deviation and verified in at least three independent experiments. (i) Normalised fluorescent intensity of DCFH‐DA in control, D‐Gal, D‐Gal+ECH and D‐Gal+ECH+Tonabersat (10 μM) groups; ***p < 0.001. (j) Normalised fluorescent intensity of GSH in control, D‐Gal, D‐Gal+ECH and D‐Gal+ECH+Tonabersat (10 μM) groups; ***p < 0.001. (k) Normalised fluorescent intensity of mitochondria in control, D‐Gal, D‐Gal+ECH and D‐Gal+ECH+Tonabersat (10 μM) groups; ***p < 0.001. (l) Normalised fluorescent intensity of SIRT1 in control, D‐Gal, D‐Gal+ECH and D‐Gal+ECH+Tonabersat (10 μM) groups. *p < 0.05; ***p < 0.001. (m) Normalised fluorescent intensity of H3K9me3 in control, D‐Gal, D‐Gal+ECH and D‐Gal+ECH+Tonabersat (10 μM) groups. **p < 0.01; ***p < 0.001. All data are expressed as the mean ± standard deviation and verified in at least three independent experiments.

To elucidate the molecular mechanisms underlying ECH's rejuvenating effects on senescent oocytes, we focused on GJA1, a protein that plays a critical role in mitochondrial regulation and resistance to OS. Additionally, SIRT1, known for its regulation of cellular lifespan and mitochondrial function, has been closely linked to oocyte quality [31]. Previous studies have demonstrated that GJA1 can increase SIRT1 expression [32], suggesting that ECH may exert its beneficial effects on oocyte health through the GJA1/SIRT1 pathway. To test this hypothesis, we used the GJA1 inhibitor Tonabersat and selected a concentration of 10 μM based on its significant impact on cumulus cell expansion and first polar body extrusion in porcine oocytes (Figure 6a,d,h). Oocytes were divided into four groups: control, D‐Gal, D‐Gal+ECH, and D‐Gal+ECH+Tonabersat (10 μM) to investigate whether ECH could enhance senescent oocyte function via the GJA1/SIRT1 pathway.

The results showed that the mitochondrial function enhanced by ECH treatment was significantly impaired when GJA1 was inhibited by Tonabersat. Similarly, ECH‐induced increases in glutathione (GSH) levels were reversed upon GJA1 inhibition, resulting in elevated OS as indicated by increased ROS levels in the inhibited group (Figure 6b,c,i,j). Further analysis revealed that ECH treatment significantly upregulated SIRT1 expression, which played a pivotal role in reducing OS and improving mitochondrial function in senescent oocytes (Figure 6e,f,k,l). However, this upregulation of SIRT1 was suppressed when GJA1 was inhibited by Tonabersat.

Additionally, histone modification associated with SIRT1 activity, H3K9me3, was significantly reduced in the Tonabersat‐inhibited group, whereas ECH treatment alone resulted in increased H3K9me3 levels (Figure 6g,m). These findings collectively indicate that ECH improves senescent oocyte function by acting through the GJA1/SIRT1 pathway, enhancing mitochondrial function, reducing OS, and regulating histone modifications.

## 4 Discussion

Oocyte aging is a key factor in reproductive aging, marked by a progressive decline in oocyte quality, increased OS, mitochondrial dysfunction and chromosomal abnormalities. These age‐related changes compromise fertility and raise the risk of pregnancy complications. Developing effective strategies to counteract oocyte aging and improve reproductive outcomes is therefore crucial. In this study, we demonstrated that ECH, a natural compound, can alleviate oocyte aging by enhancing mitochondrial function, reducing OS, and improving chromosomal stability in D‐Gal‐induced aging oocytes.

D‐Gal has been widely recognised for its ability to induce OS and is frequently used as a model of senescence in various cells [33, 34]. Consistent with previous studies, our results confirmed that D‐Gal‐induced porcine oocytes exhibited meiotic defects, including abnormal spindle formation and reduced F‐actin levels, indicating impaired oocyte maturation [35]. ECH treatment effectively reversed these abnormalities, restoring spindle polarity and enhancing F‐actin integrity. This finding underscores the role of ECH in improving cytoskeletal stability, which is crucial for accurate meiotic progression.

Mitochondria play a central role in the aging process of oocytes, with changes in their function directly impacting oocyte quality and reproductive potential [36]. Studies have shown that mitochondrial transplantation can improve oocyte quality and reproductive outcomes, emphasising the importance of mitochondrial health [37]. In our model, D‐Gal‐induced oocytes showed a significant decrease in MMP and irregular mitochondrial distribution, indicating mitochondrial depolarisation and dysfunction. ECH treatment restored MMP and normalised mitochondrial distribution, demonstrating its protective effect on mitochondrial integrity. Additionally, ECH regulated mitochondrial dynamics by downregulating fission‐related proteins such as DRP1 and FIS1, thereby balancing mitochondrial fission and fusion. These findings highlight ECH's role in stabilising mitochondrial function, which is crucial for combating oocyte aging.

Approximately 90% of intracellular ROS originates from mitochondrial aerobic respiration, where normal levels are essential for follicle development and survival [38, 39]. However, aging‐associated mitochondrial decline leads to increased electron leakage in the electron transport chain, resulting in elevated ROS production [40]. Excessive ROS overwhelms cellular antioxidant defences, causing OS and damage to proteins, lipids, and DNA [41]. Our study showed that D‐Gal‐induced aged oocytes exhibited elevated ROS levels and increased expression of the DNA damage marker γ‐H2AX, reflecting genomic instability caused by OS. ECH treatment significantly reduced ROS levels and γ‐H2AX expression, suggesting its potential role in mitigating OS and enhancing genomic stability. Furthermore, ECH promoted the recovery of lipid metabolism and lysosomal function, increased lipid droplet accumulation, improved cellular waste clearance, and helped maintain metabolic balance and cellular homeostasis.

Autophagy is an important mechanism that allows cells to cope with stress, but it often becomes abnormally activated or suppressed during aging [42, 43]. In our study, D‐Gal treatment increased autophagy in oocytes, which is consistent with previous studies showing that D‐Gal activates autophagy as an adaptive response to stress [44, 45]. ECH was able to restore autophagy to normal levels and maintain a moderately active state by regulating the PINK1/Parkin signalling pathway, thereby avoiding the detrimental effects of excessive or insufficient autophagy on cell health. By normalising mitochondrial autophagy, ECH further supported mitochondrial function and intracellular stability.

Transcriptomic analysis revealed that ECH regulates key genes associated with mitochondrial function and redox balance, particularly GJA1, also known as Connexin 43 (CX43). Recent research has demonstrated that GJA1 is not only localised to the plasma membrane but also expressed on mitochondria, where it plays a vital role in maintaining mitochondrial homeostasis [28, 46, 47]. Specifically, GJA1 contributes to maintaining MMP and enhances ATP generation, which protects cells against OS [29, 30]. Furthermore, GJA1 has been identified as an important regulator of mitochondrial fission and mitophagy, which are critical processes for maintaining mitochondrial function and cellular homeostasis [48]. Molecular docking and biolayer interferometry confirmed the strong binding affinity between ECH and GJA1. Further studies demonstrated that GJA1 might mediate ECH's protective effects through the SIRT1 signalling pathway, which is involved in mitochondrial regulation and OS resistance. OS is a key factor in oocyte senescence and triggers mitochondrial damage and apoptosis [49, 50]. Given that SIRT1 activity declines with age and plays a crucial role in reducing ROS and preserving oocyte quality, our results suggest that GJA1 may exert antioxidant effects in aging oocytes by promoting SIRT1 expression. Inhibition of GJA1 significantly weakened ECH's ability to improve mitochondrial function and reduce OS, confirming the essential role of the GJA1/SIRT1 pathway in ECH's anti‐aging effects and suggesting the possibility that ECH acts as a SIRT1 activator. Additionally, ECH enhanced genome stability and anti‐aging outcomes by modulating histone H3K9 methylation levels. These findings provide a mechanistic insight into how ECH alleviates oocyte aging and suggest that targeting the GJA1/SIRT1 axis may serve as a promising therapeutic strategy to improve oocyte quality and reproductive outcomes in women of AMA.

In conclusion, this study provides convincing evidence that ECH can effectively combat oocyte aging through multiple mechanisms. By restoring mitochondrial function, alleviating OS, regulating cellular homeostasis and promoting genomic stability, ECH offers a promising strategy to enhance oocyte quality and potentially delay the onset of reproductive aging. The identification of the GJA1/SIRT1 pathway as a key mediator of ECH's effects further elucidates its molecular mechanisms and opens new avenues for targeted therapeutic interventions aimed at mitigating age‐related fertility decline.

## Author Contributions

**Liuqing Yang:** methodology, investigation, project administration, funding acquisition, and writing – review and editing. **Xinle Lai:** visualization, validation, investigation, data curation. **Fangxuan Lin:** writing – original draft, visualization, data curation. **Nan Shi:** writing – original draft, visualization, data curation. **Xinya Xu:** visualization, data curation. **Heng Wang:** visualization, data curation. **Xiaotian Li:** data curation. **Dan Shen:** investigation. **Haimo Qian:** formal analysis. **Xin Jin:** formal analysis. **Jiayi Chen:** writing – original draft. **Zhongwei Huang:** review and editing, supervision. **Xing Duan:** writing – review and editing, validation, supervision, project administration, methodology, conceptualization. **Qin Zhang:** supervision, project administration, funding acquisition.

## Ethics Statement

All experimental protocols were conducted in strict accordance with the ethical guidelines of the Institutional Animal Care and Use Committee of Zhejiang Chinese Medical University, Zhejiang, China.

## Conflicts of Interest

The authors declare no conflicts of interest.

## Supporting information


**Figure S1.** ECH improves early cleavage of porcine parthenogenetic embryos impaired by D‐Gal treatment. (a) Representative images of 2‐cell and 4‐cell stage embryos at 24 and 48 h post‐activation in the Control, D‐Gal, and D‐Gal + ECH groups. Scale bar = 100 μm. (b) Cleavage rates (%) of embryos at the 2‐cell and 4‐cell stages. D‐Gal treatment significantly reduced cleavage rates compared to the control, while ECH supplementation effectively improved the cleavage rate. Data are presented as mean ± SEM. *p < 0.05; **p < 0.01; ***p < 0.001.

## Data Availability

The data that support the findings of this study are available on request from the corresponding author. The data are not publicly available due to privacy or ethical restrictions.
