# Supplementary figures and images for: Revitalising Aging Oocytes: Echinacoside Restores Mitochondrial Function and Cellular Homeostasis Through Targeting GJA1/SIRT1 Pathway
Source: Cell Prolif. 2025 Apr 18;58(10):e70044. doi: 10.1111/cpr.70044 (PMC12508679; doi:10.1111/cpr.70044)

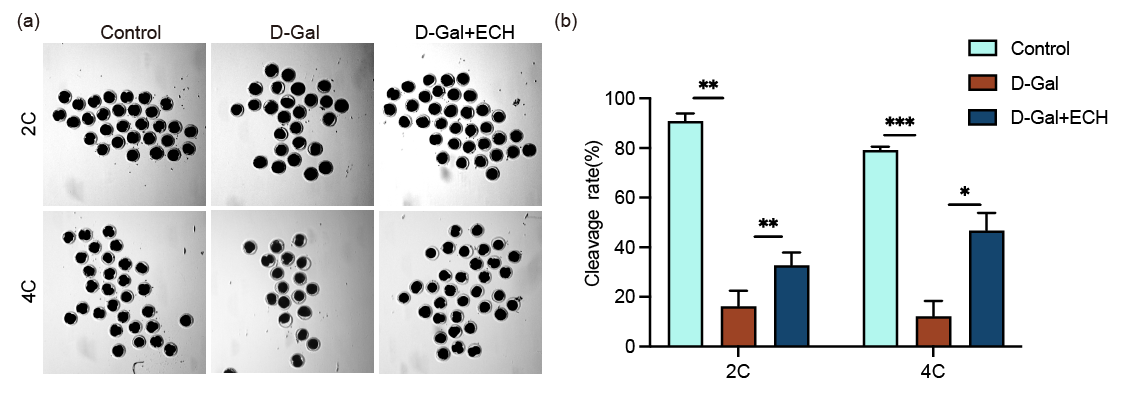

Supplement: Supplementary file 1 — Figure S1. ECH improves early cleavage of porcine parthenogenetic embryos impaired by D‐Gal treatment. (a) Representative images of 2‐cell and 4‐cell stage embryos at 24 and 48 h post‐activation in the Control, D‐Gal, and D‐Gal + ECH groups. Scale bar = 100 μm. (b) Cleavage rates (%) of embryos at the 2‐cell and 4‐cell stages. D‐Gal treatment significantly reduced cleavage rates compared to the control, while ECH supplementation effectively improved the cleavage rate. Data are presented as mean ± SEM. *p < 0.05; **p < 0.01; ***p < 0.001. [file CPR-58-e70044-s001.png]
